# Intestinal Damage Determines the Inflammatory Response and Early Complications in Patients Receiving Conditioning for a Stem Cell Transplantation

**DOI:** 10.1371/journal.pone.0015156

**Published:** 2010-12-20

**Authors:** Walter J. F. M. van der Velden, Alexandra H. E. Herbers, Ton Feuth, Nicolaas P. M. Schaap, J. Peter Donnelly, Nicole M. A. Blijlevens

**Affiliations:** 1 Department of Hematology, Radboud University Nijmegen Medical Center, Nijmegen, the Netherlands; 2 Department of Epidemiology, Biostatistics and Health Technology Assessment, Radboud University Nijmegen Medical Center, Nijmegen, the Netherlands; Charité-University Medicine Berlin, Germany

## Abstract

**Background:**

Stem cell transplantation (SCT) is still complicated by the occurrence of fever and inflammatory complications attributed to neutropenia and subsequent infectious complications. The role of mucosal barrier injury (MBI) of the intestinal tract therein has received little attention.

**Methods:**

We performed a retrospective analysis in 163 SCT recipients of which data had been collected prospectively on intestinal damage (citrulline), inflammation (C-reactive protein), and neutrophil count. Six different conditioning regimens were studied; 5 myeloablative (MA) and 1 non-myeloablative (NMA). Linear mixed model multivariate and AUC analyses were used to define the role of intestinal damage in post-SCT inflammation. We also studied the relationship between the degree of intestinal damage and the occurrence of early post-SCT complications.

**Results:**

In the 5 MA regimen there was a striking pattern of inflammatory response that coincided with the occurrence of severe intestinal damage. This contrasted with a modest inflammatory response seen in the NMA regimen in which intestinal damage was limited. With linear mixed model analysis the degree of intestinal damage was shown the most important determinant of the inflammatory response, and both neutropenia and bacteremia had only a minor impact. AUC analysis revealed a strong correlation between citrulline and CRP (Pearson correlation r = 0.96). Intestinal damage was associated with the occurrence of bacteremia and acute lung injury, and influenced the kinetics of acute graft-versus-host disease.

**Conclusion:**

The degree of intestinal damage after myeloablative conditioning appeared to be the most important determined the inflammatory response following SCT, and was associated with inflammatory complications. Studies should explore ways to ameliorate cytotoxic therapy-induced intestinal damage in order to reduce complications associated with myeloablative conditioning therapy.

## Introduction

Treating patients with hematological malignancies by use of a hematopoietic stem cell transplantation (SCT) is still complicated by the occurrence of infections and inflammatory complications including sepsis, acute lung injury, and graft-versus-host disease (GvHD). Historically the focus was on neutropenia and fever (“febrile neutropenia”) and its relation to infections [1]. However, a substantial number of SCT recipients develop fever unrelated to infection (“unexplained fever”) [2], resulting from other causes including cytotoxic therapy-induced mucosal barrier injury (MBI) [3], [4].

Many studies have shown associations between the magnitude of the C-reactive protein (CRP) response and cytokine release and post-SCT complications [5]–[9], and these complications might therefore best be regarded as manifestations of a systemic inflammatory response syndrome (SIRS) [8]. Other studies have shown that infections may contribute to non-infectious complications including acute GvHD [10], [11]. However, few if any of these studies addressed the role of MBI *per se* as an isolated cause of inflammation and risk factor for infections, nor its role in the pathogenesis of inflammatory complications. Animal models have enhanced our understanding of the inflammatory processes that take place in the intestine following chemotherapy [12]–[14], and in the clinical setting of SCT the relationship between intestinal damage and the inflammatory response has become better appreciated [4], [15]. Mucosal damage and deregulated host-microbial interactions have also been shown to contribute to SIRS and post-SCT complications such as acute GvHD [16], [17]. Therefore, we hypothesized that intestinal damage could be the most important determinant of early SIRS following conditioning with chemotherapy and radiotherapy and that the degree of damage correlates with the occurrence of post-SCT complications.

Studying damage to the gastro-intestinal tract during SCT remains difficult, since it remains hidden and hitherto only indirect and non-specific tests were available [18], [19]. Measuring serum or plasma citrulline levels provides a more direct and specific way of determining intestinal damage of certain conditions that are accompanied by gut failure [20]. Recently, citrulline-based assessment of intestinal damage has also shown to be objective, reproducible, specific and reliable in the setting of SCT [21], [22]. To test our hypothesis we studied the relationship between the magnitude of the inflammatory response and the degree of intestinal damage as measured by citrulline, the duration of neutropenia, and the occurrence of bacteremia. To achieve this we selected recipients of a SCT for which 5 cohorts of patients had been given different myeloablative (MA) conditioning regimens and a single cohort had received a non-myeloablative (NMA) conditioning regimen. We also investigated whether we could determine a relationship between the degree of intestinal damage and the occurrence of early post-SCT complications.

## Materials and Methods

### Study population

This was a retrospective analysis of 163 patients who had received an autologous or allogeneic SCT in our hospital from May 2004 to December 2007. Plasma had been collected prospectively and stored for later analysis of citrulline, but other data including CRP, temperature, and clinical and microbiological infections had been prospectively gathered from the day of starting chemotherapy. Patients had given their informed consent to the prospective collection of data and plasma samples for investigational use. The local ethics committee (CMO Regio Arnhem-Nijmegen) judged that no formal approval for this study was necessary regarding the fact that data were used anonymously and the analysis would not reveal results harming contributing patients.

### Conditioning regimens and stem cell transplantation

The MA and NMA regimens are depicted in Table 1. All patients received a stem cell graft on the day scheduled. Autologous SCT grafts contained at least 2.0×10^6^ CD34+ cells per kg, and allogeneic SCT partially T cell-depleted grafts contained on average 3.0×10^6^ CD34+ cells per kg and 0.5×10^6^ CD3+ cells per kg.

**Table 1:** Conditioning regimens.

Regimen	Doses	Frequency	Days	Type of conditioning	Type of SCT, day
HDM-Melphalan	100 mg/m^2^	Od	1, 2	MA	Autologous, day 4
BEAM-Carmustine (BCNU)-Etoposide.-Cytarabine-Melphalan	300 mg/m^2^100 mg/m^2^100 mg/m^2^140 mg/m^2^	OdBdBdOd	12-52-56	MA	Autologous, day 7
Ida-Cyclo-TBI:-Idarubicine-Cyclophosphamide-TBI	42 mg/m^2^60 mg/kg4.5 Gy	Infusion over 48 hOdOd	17, 811, 12	MA	AllogeneicMatched related donor, day 13
Cyclo-ATG-TBI-Cyclophosphamide-Thymoglobuline-TBI	60 mg/kg2 mg/kg4.5 Gy	OdOdOd	1, 23-67, 8	MA	AllogeneicMatched unrelated donor, day 9
Cyclo-TBI:-Cyclophosphamide-TBI	60 mg/kg4.5 Gy	OdOd	1, 25, 6	MA	AllogeneicMatched related donor, day 7
Cyclo-Flu:-Cyclophosphamide-Fludarabine	1200 mg/m^2^30 mg/m^2^	OdOd	1-41-4	NMA	AllogeneicMatched related donor, day 7

Abbreviations: od; once daily, bd; two times daily, TBI = total body irradiotion, MA = myeloablative, NMA = non-myeloablative.

### Treatment protocol

All patients were treated according to the same protocol, which has been described earlier [23]. GvHD prophylaxis consisted of cyclosporine only. Anti-microbial prophylaxis consisted of ciprofloxacin and valacyclovir. Fever was defined by a single axillary temperature ≥38.5°C. At the onset of fever 40 mL of peripheral blood was obtained for culture together with 10 mL from each lumen of the catheter, patients were examined for any sign of local infection, and empirical therapy with ceftazidime was started [24].

Neutropenia was defined as an ANC≤0.5×10^9^/L, and the duration and depth was recorded. CRP levels (mg/L) were determined every day and the maximum CRP (CRP_max_) recorded. Plasma citrulline was determined to estimate intestinal damage before the start of conditioning and 3 times weekly thereafter until discharge. Citrulline concentrations (µmol/L) were measured by a standard procedure for determining amino acids using high-performance liquid chromatography [21]. Citrulline levels below 10 µmol/L were deemed to indicate hypocitrullinemia, and were regarded as reflecting severe intestinal damage [20].

### Definition of stem cell transplantation complications

Clinical and microbiologically defined infections were defined according to the Consensus definitions of Immunocompromised Host Society [2]. A blood culture was considered to represent bacteremia if one or more bottles yielded a microorganism, except in the case of coagulase-negative staphylococci (CoNS), which required recovery of the same strain from two separate positive blood cultures [24]. The incidence of bacteremia that occurred on the day of fever was documented and compared among the regimens. Invasive fungal diseases were scored according to the EORTC/MSG consensus guidelines [25]. Acute lung injury (ALI) was defined according to current guidelines [26]. Acute GvHD, GvHD occurring the first 100 days after SCT, was graded according to the criteria of *Przepiorka et al.* [27]. Early mortality related to SCT complications was defined as any death occurring within 30 days following SCT (day +30), but unrelated to the underlying disease.

### Data analysis

We employed descriptive statistics for fever, neutrophil count, CRP levels, and citrulline levels which were expressed as mean values together with the 95% confidence interval (Table 2). As citrulline was measured three times weekly, the real nadir might have been attained between two measurements and hence was likely missed. To compensate for this and study the true length of time in which citrulline levels were below 10 µmol/L we modeled the course of citrulline as a function of time during the first 30 days by developing a linear mixed model using first, second, third and fourth power of time as fixed factors to predict the citrulline levels after taking into account the within-person correlations by incorporating a random patient intercept. To describe the relationship of CRP to the neutrophil count, intestinal damage (citrulline concentration) and bacteremia we used several linear mixed models for the first 30 days with random patient effect and the logarithmic transformed CRP (*log* CRP) as the outcome variable.

**Table 2:** General characteristics.

Conditioning	HDM(N = 56)	BEAM(N = 21)	Ida-Cyclo-TBI(N = 28)	Cyclo-ATG-TBI(N = 34)	Cyclo-TBI (N = 10)	Cyclo-Flu(N = 14)
Age, mean (range), years	56 (33-65)	47 (18-65)	46 (18-64)	43 (20-58)	50 (38-59)	54 (39-65)
Gender: M/F	35/21	17/4	13/15	21/13	8/2	10/4
Diagnoses:-MM-NHL/CLL-AML-ALL-MDS-CML/MPD	56 (100%)-----	-21 (100%)----	-7 (25%)12 (42.5%)3 (10.5%)6 (22%)-	-13 (38%)8 (23.5%)4 (11.75%)4 (11.75%)5 (15%)	8 (80%)2 (20%)---	14 (100%)-----
Type of conditioning	MA	MA	MA	MA	MA	NMA
Type of SCT	Autologous	Autologous	Matched sibling allogeneic	Matched unrelated allogeneic	Matched sibling allogeneic	Matched sibling allogeneic
Fever (axillary temperature ≥ 38.5^0^C)	88.0%	90.5%	100%	Early: 73.5%Late: 100%	100%	Early: 28.6%Late: 78.6%
Fever, day from start conditioning, mean (95%CI)	11.8 (11.4-12.2)	13.0 (12.2-13.9)	12.9 (12.2-13.6)	4.1 (3.7-4.5)14.1 (13.0-15.1)	13.4 (12.0-14.8)	3.0 (1.7-4.3)16.0 (14.4-17.5)
Neutrophils <0.5 x10^9^/L, days (95%CI)	8.4 (8.0-8.7)	9.5 (8.5-10.5)	15.8 (14.6-17.0)	15.5 (14.6-16.4)	11.1 (9.8-12.4)	12.4 (10.8-14.1)
Citrulline <10 µmol/L, number of patients, (%)	51 (91%)	21 (100%)	26 (93%)	30 (88%)	10 (100%)	4 (29%)
Measurements with citrulline <10 µmol/L, mean (95%CI)*#	3.5 (3.2-3.9)	4.7 (4.0-5.3)	6.2 (4.6-7.8)	4.8 (4.0-5.7)	3.5 (2.4-4.6)	3.0 (1.1-4.9)
Observed citrulline nadir µmol/L, mean (95%CI)*	6.0 (5.4-6.6)	4.3(3.5-5.1)	4.6 (3.9-5.3)	5.6 (4.9-6.3)	5.6 (4.2-7.0)	10.8 (8.9-12.6)
Citrulline <10 µmol/L, days, mean (95%CI)&#	7.9 (7.1-8.7)	11.2 (9.6-12.9)	17.7 (15.6-19.8)	14.6 (13.3-15.9)	11.0 (7.9-14.1)	7.5 (5.4-9.6)
Citrulline nadir µmol/L, mean (95%CI)&	6.5 (5.7-7.2)	4.9 (3.7-6.0)	4.5 (3.5-5.7)	7.0 (6.1-7.9)	6.6 (5.1-8.1)	12.4 (10.2-14.6)
CRP_max_ (mg/L), mean (95%CI)	163 (136-189)	202(160-246)	257 (222-291)	188 (162-213)	211 (154-269)	66 (38-95)

Characteristics of patients, stem cell transplantation and general outcome measures of intestinal damage (citrulline), inflammation (CRP and fever), and neutropenia (neutrophil count ≤0.5×10^9^/L) for each conditioning regimen.

*Citrulline was measured 3 times weekly.

# Only those patients included with citrulline levels below 10 µmol/L.

& Based on estimated values. MA = myeloablative, NMA = non-myeloablative, CRP = C-reactive protein, MM = multiple myeloma, NHL = non-Hodgkin lymphoma, CLL = chronic lymphatic leukemia, AML/ALL = acute myeloid and lymphatic leukemia, MDS = myelodysplastic syndrome, CML/MPD = chronic myeloid leukemia/myeloproliferative disease.

To assess the impact of conditioning on intestinal damage and CRP we performed an area under the curve (AUC) analyses. Per patient, the CRP_AUC_ was defined as the sum of the 30 estimated CRP values, resulting from a piecewise linear mixed model which uses a linear time component for day 1–10 and a cubic time component for day 11–30. The conditioning regimen and the interactions between the particular regimen and time were also part of this model that also accounted for within person correlations by virtue of a random intercept. Likewise, the citrulline_AUC_ per patient was defined as the sum of the 30 estimated 10/citrulline values. We used the citrulline levels estimated by the linear mixed model described above and transformed these values into 10 times the inverse of the estimated value.

Comparisons between the impact of the different conditioning regimens on neutropenia, CRP_AUC_ and citrulline_AUC_ were studied using the Kruskal-Wallis test. The correlation between the degree of neutropenia and CRP_AUC_ versus citrulline_AUC_ and CRP_AUC_ was studied by Pearson correlation over the different regimens.

Comparison of the mean onset of acute GvHD between the different regimens was done using one-way ANOVA. Comparison of the incidence of ALI in relation to OVS between the different regimens was done using the χ^2^-test.

Analyses were performed using SAS 8.2 and a *P*-value of <0.05 was considered to indicate statistical significance.

## Results

### Study population and patient characteristics

Seventy-seven (77) patients received an autologous and 86 an allogeneic SCT (Table 2). All but 14 patients received MA conditioning. Autologous SCT was performed for patients with multiple myeloma and non-Hodgkin lymphoma, but allogeneic SCT was employed for a greater variety of diagnoses including acute and chronic lymphatic and myeloid leukemia and myelodysplastic syndrome. NMA conditioning was employed to prepare patients who had received an autologous SCT 4–6 months earlier for MM.

### Intestinal damage

**Figure 1:**
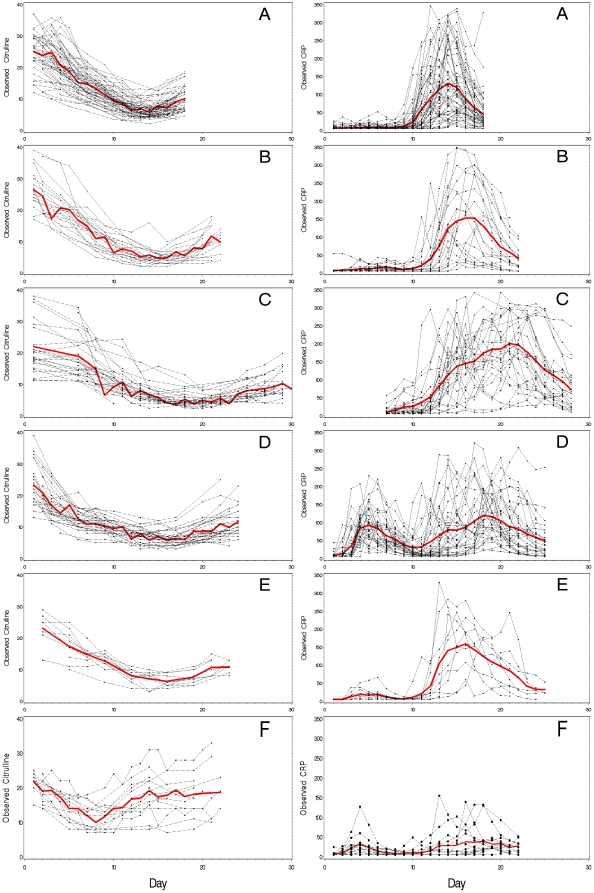
Course of citrulline and CRP in time after start of conditioning. Five MA and one NMA conditioning regimens are shown; A = HDM, B = BEAM, C = Ida-Cyclo-TBI, D = Cyclo-ATG-TBI, E = Cyclo-TBI, F = Cyclo-Flu. Observed values (•), mean values (○).

**Figure 2:**
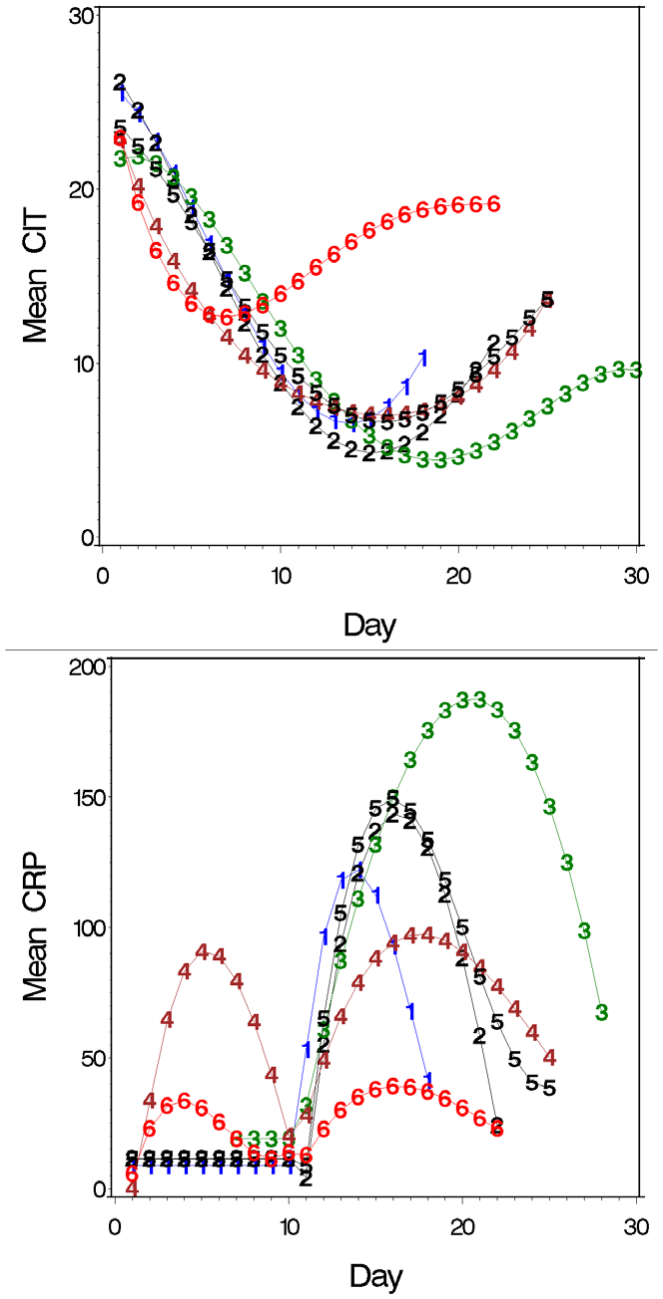
Summary of the time course of citrulline (A) and CRP (B) for all 6 regimens. Day 1 is the day of start of conditioning. To correct for unobserved citrulline and CRP values we modeled the course of citrulline and CRP as described in methods. 1 = HDM, 2 = BEAM, 3 = Ida-Cyclo-TBI, 4 = Cyclo-ATG-TBI, 5 = Cyclo-TBI, 6 = Cyclo-Flu. Mean CRP in mg/L, mean citrullline in µmol/L.

MA conditioning was associated with severe and prolonged intestinal damage shown by a rapid decline in citrulline to <10 µmol/L, a mean of 10 days after starting chemotherapy. The mean nadir of citrulline was 4.5–7.0 µmol/L, and hypocitrullinemia lasted for more than one week in most patients (Figure 1A–E, Figure 2A, Table 2). Hypocitrullinemia was most pronounced in patients receiving idarubicin in their conditioning, lasting approximately 18 days. In contrast, an early and short drop of citrulline level was noticed for NMA conditioning, but hypocitrullinemia was not evident for most patients (Figure 1F, Figure 2A, Table 2).

### Inflammatory response measured by C-reactive protein and fever

The course of CRP during SCT of the different conditioning regimens is illustrated in Figure 1 and Figure 2B. Within each type of MA conditioning, patients showed similar patterns of inflammatory response, although there was some variation in the precise onset, peak and resolution of CRP levels. Those without bacteremia did not have a different course when compared to those with; although in general CRP levels were lower (data not shown). As for intestinal damage, the CRP response was highest in patients receiving idarubicin. Resolution of inflammation occurred with engraftment and restoration of the intestinal damage defined by rising citrulline levels. In Cyclo-ATG-TBI conditioning the first peak of CRP was related to ATG induced lymphocyte depletion and cytokine release, but the second peak resembled that seen for the other MA regimens. Also some patients treated with Cyclo-TBI and BEAM had an early peak in CRP during conditioning, which was probably related to chemotherapy induced cytokine release.

Only a moderate inflammatory response occurred after NMA (Figure 1F and Figure 2B). Also, the timing was different when compared to MA regimens with a peak occurring early during conditioning and a second peak much later. The latter occurred during engraftment and thereby resembled to some extent the inflammatory complication designated engraftment syndrome [28].

Virtually every patient who had received MA developed fever as did 80% of those given NMA conditioning. Some patients receiving Cyclo-ATG-TBI and Cyclo-Flu also experienced an early episode with fever during conditioning (25 and 4 patients, respectively). In MA regimens fever occurred on days 12–14, 2–3 days after CRP had become elevated (Figure 1, Table 2). By contrast, fever occurred late during engraftment at a mean of day 16 after starting NMA conditioning.

### Relation intestinal damage to inflammation

In MA conditioning CRP levels started to increase from 10–11 following the start of conditioning which coincided with the development of hypocitrullinemia (Figure 1A–E and Figure 2). The peak of the inflammatory response coincided with the nadir of citrulline levels. Although interindividual differences existed the occurrence of inflammation was related to the development of intestinal damage in almost every patient.

**Figure 3:**
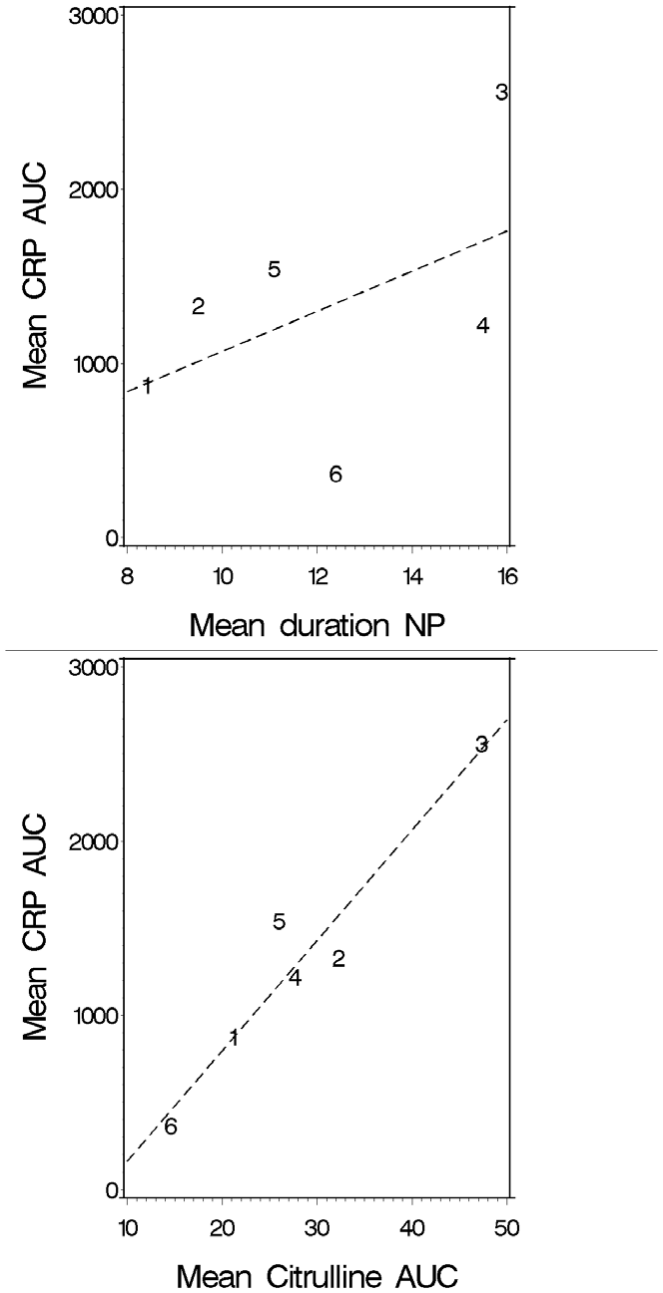
Pearson correlation between the mean degrees of neutropenia (NP in days) and inflammation (CRP_AUC_) versus intestinal damage (Citrulline_AUC_) and inflammation (CRP_AUC_) over the different regimens. 1 = HDM, 2 = BEAM, 3 = Ida-Cyclo-TBI, 4 = Cyclo-ATG-TBI, 5 = Cyclo-TBI, 6 = Cyclo-Flu.

Additional AUC analysis was used to grade the impact of conditioning on intestinal damage and CRP. There were significant differences in both CRP_AUC_ and citrulline_AUC_ between the various conditioning regimens (Kruskal-Wallis P<0.001), except between BEAM, Cyclo-TBI, and Cyclo-ATG-TBI. Interestingly, a very strong correlation between the degree of intestinal damage and the inflammatory response was seen for the different regimens (Pearson correlation 0.96, Figure 3). By contrast, there was only a weak correlation between the duration of neutropenia and inflammation (Pearson correlation 0.48, Figure 3).

In univariate linear mixed model analysis, 10/citrulline, the type of conditioning regimen, neutropenia and bacteremia were significantly associated with *log* CRP. In multivariable analyses only citrulline and type of conditioning regimen remained important.

### Stem cell transplantation complications

#### Bacteremia

There was a significant difference between MA and NMA regarding bacteremia on the day of fever (Table 3) with up to 85% of patients experiencing bacteremia predominantly due to oral viridans streptococci (OVS) and CoNS after MA conditioning, compared with none of those receiving NMA (*P*<0.001). OVS was recovered with CoNS in 20/55 (36%) of cases. A minority of patients experiencing a bacteremia with CoNS on the day of fever had any clinical or radiological signs of a CVC related tunnel infection or thrombophlebitis at the same time (5/55 (9%)).

**Table 3:** Stem cell transplantation complications.

SCT complications	HDM(N = 56)	BEAM(N = 21)	Ida-Cyclo-TBI(N = 28)	Cyclo-ATG-TBI(N = 34)	Cyclo-TBI (N = 10)	Cyclo-Flu(N = 14)
Bacteremia on day of fever-OVS-CoNS-Other	2922 (39.3%)12 (21.4%)2	106 (28.5%)7 (33.3%)-	187 (25%)12 (46.5%)1	2913 (38%)21 (65%)1	65 (50%)3 (20%)-	0---
Concomitant OVS/CoNS	7	3	2	6	2	NA
Candidemia	1	0	3	1	0	0
Clinically defined infection:-Phlebitis superficial vein.-Tunnel infection/infected thrombosis-Pneumonia.-Probable/Proven IA.	5-14-	5-32-	9-521	7-6-1	3-111	31-2-
ALIALI following OVS bacteremia	4/56 (7.1%)3/22 (13.6%)	2/21 (9.5%)1/6 (16.7%)	6/28 (21.5%)4/7 (57.1%)	4/34 (11.8%)4/13 (30.8%)	2/10 (20%)2/5 (40%)	0
Early mortality (day +30 post SCT)-ALI.-Acute GvHD	0--	11-	211	1-1	211	0--
aGvHD I-IV, all (N, %)-Grade II-IV-Grade III-IV	NA	NA	13/28 (46%)8 (28.5%)2 (7%)	12/34 (28%)7 (20.5%)2 (6%)	6/10 (60%)3 (30%)1 (10%)	6/14 (43%)4 (28.5%)-
Onset from day SCT, mean (95%CI)	NA	NA	26 (16-36)	46 (29-63)	23 (19-27)	40 (19-61)

OVS = oral viridians streptococci, CoNS = coagulase-negative staphylococci, IA = invasive *Aspergillosis*, ALI = acute lung injury, acute GvHD = acute graft-versus-host disease. Grading of acute GvHD was done according to the criteria of *Przepiorka et al.* [27] and probable/proven IA was defined according to EORTC/MSG consensus definitions [25].

#### Acute lung injury

The overall incidence of ALI was 18/163 (11%), with 14/18 (78%), being associated with OVS bacteremia. Conversely, ALI affected 14/53 (26.4%) patients with OVS bacteremia. However, this incidence varied significantly between conditioning regimens and was related to the severity of intestinal damage as ALI occurred in 3/22 (13.6%), 4/13 (30.8%) and 4/7 (57.1%) in patients receiving HDM, Cyclo-ATG-TBI, and Ida-Cyclo-TBI conditioning, respectively (*P* = 0.03) (Table 3).

#### Acute graft-versus-host disease

No differences were seen in the total incidence of acute GvHD, although there were no cases of acute GvHD III-IV in the group with NMA; with only skin acute GvHD being encountered. However, there was a significant difference in the onset of acute GvHD. In Ida-Cyclo-TBI and Cyclo-TBI, despite receiving a partially T-cell-depleted graft, acute GvHD occurred early with a mean onset on day +25 post SCT. In both Cyclo-ATG-TBI and Cyclo-Flu the onset was delayed, with a mean onset on day +46 and +40 post SCT, respectively (*P* = 0.02).

#### Early mortality

Overall, early mortality was low 6/163 (3.7%), and related to ALI and acute GvHD, and all but one death occurred following MA conditioning for an allogeneic SCT.

## Discussion

In this study we show the course and extent of intestinal damage and inflammatory responses following various conditioning regimens used to prepare for a hematopoietic SCT. There was a striking pattern of inflammatory response coinciding with the occurrence of severe intestinal damage for patients receiving MA conditioning, defined by hypocitrullinemia [20]. Moreover, the degree of intestinal damage seemed the most important determinant of inflammation and was highly correlated with the magnitude of the inflammatory response measured by CRP. Neither neutropenia nor bacteremia had a major impact on this. The close relationship between intestinal damage and inflammation was further underscored by the fact that NMA resulted in only a modest inflammatory response with a completely different time course, and the virtual absence of severe intestinal damage. Consequently, intestinal damage appears the primary determinant of inflammation following myeloablative conditioning with chemo- and radiotherapy in the setting of autologous and allogeneic SCT.

While there are limitations associated with retrospective analysis and the potential for bias resulting from selection, the relationship of intestinal damage to SCT complications was remarkable. As expected, there was a significant difference in occurrence of bacteremia between those receiving MA and NMA conditioning [29]. The similar duration of neutropenia and the marked difference in intestinal damage, suggest that the gut may have been the origin of bacteremia [30]. Moreover, most pathogens recovered from blood cultures are known residents of the gut [31], [32]. Notably, a considerable proportion of patients had bacteremia with both CoNS and OVS, which was probably due to simultaneous intestinal translocation [31].

A strong relationship has been found between the occurrence of OVS bacteremia and septic shock and ALI in neutropenic patients, which was explained by virulence factors and pulmonary cytotoxicity of chemotherapy [33]–[35]. Interestingly, we saw that in patients with OVS bacteremia the incidence of ALI was related to the degree of intestinal damage. It is known that barrier dysfunction facilitates bacterial translocation, but intestinal damage also seems to determine the resulting inflammatory response. This was confirmed in our linear mixed model, which showed citrulline but not bacteremia related to the CRP response. Although ALI seems directly associated with OVS bacteremia this might be only coincidental, as both complications are consequences of severe intestinal damage. So intestinal damage ‘primes’ the immune system with subsequent aggravated cytokine release following activation of pattern recognition receptors from translocating microbial motifs [13].

This ‘priming’ of the immune system also applies to the occurrence acute GvHD in which the role of intestinal damage has been acknowledged [16]. Although we found no apparent differences in the citrulline and CRP levels between patients with and without acute GvHD within any given regimen, between regimens there was a clear difference in the kinetics of acute GvHD. In addition severe acute GvHD did not develop after NMA, as opposed to 6–10% after MA, and GvHD of liver or gut did not occur. The early onset of acute GvHD after Ida-Cyclo-TBI and Cyclo-TBI suggests that the tissue inflammation resulting from the profound intestinal damage, contributes to the accelerated allo-reactive T-cell response [36], even in the setting of partial T cell-depletion. The delay in onset of acute GvHD in patients conditioned with ATG results from additional *in vivo* T cell-depletion creating a ‘time-window’ between the intestinal damage induced inflammation and T cell recovery. After NMA we also saw a delay in the onset of acute GvHD, which is in accordance with previous data from studies in mice [37] and humans [38]. This altered kinetics of acute GvHD was, at least in part, related to the absence of significant intestinal damage and tissue inflammation. Differences in the kinetics of acute GvHD in NMA have been largely attributed to alterations in antigen presenting cell chimerism, T cell chimerism and regulatory T cell activation [37], but our data underscore the role conditioning-induced intestinal damage plays in the complex pathogenesis of acute GvHD.

Several studies have shown correlations between CRP and the occurrence of SCT complications but they all used different cut-off values [5], [7]. CRP is not a specific marker since chemotherapy and ATG and, the process of engraftment itself, elicit inflammatory responses. Hence it is not possible to identify who is at risk or when that risk might occur. Citrulline could provide an alternative, because it is a specific marker of enterocyte mass, which decreases only when there is intestinal damage. Furthermore, citrulline levels correspond with the inflammatory responses following MA conditioning, and more importantly with SCT complications. Clearly it is necessary to confirm the predictive value of citrulline for individual patients and to define cut-off values more precisely. Classifying other conditioning regimens, by means of measuring citrulline can already help determine the need for antimicrobial prophylaxis, hospital care, and the use of anti-inflammatory treatment.

Given the role of intestinal MBI in complications after SCT studies should explore ways to ameliorate cytotoxic therapy-induced intestinal damage in order to reduce inflammatory complications associated with myeloablative conditioning therapy.
